# Exploring the gut microbiota in patients with pre-diabetes and treatment naïve diabetes type 2 - a pilot study

**DOI:** 10.1186/s12902-023-01432-0

**Published:** 2023-08-21

**Authors:** Kristin Gravdal, Katrine H. Kirste, Krzysztofa Grzelak, Graceline Tina Kirubakaran, Philippe Leissner, Adrien Saliou, Christina Casèn

**Affiliations:** 1 Genetic Analysis AS, Ulvenveien 80B, 0581 Oslo, Norway; 2 BIOASTER Microbiology Technology Institute, 40 Avenue Tony Garnier, 69007 Lyon, France

**Keywords:** Type 2 diabetes, Prediabetes, Bacterial profiling, Microbiota signatures, 16S rRNA bacterial gene

## Abstract

**Background:**

Compared to their healthy counterparts, patients with type 2 diabetes (T2D) can exhibit an altered gut microbiota composition, correlated with detrimental outcomes, including reduced insulin sensitivity, dyslipidemia, and increased markers of inflammation. However, a typical T2D microbiota profile is not established. The aim of this pilot study was to explore the gut microbiota and bacteria associated with prediabetes (pre-T2D) patients, and treatment naïve T2D patients, compared to healthy subjects.

**Methods:**

Fecal samples were collected from patients and healthy subjects (from Norway). The bacterial genomic DNA was extracted, and the microbiota analyzed utilizing the bacterial 16S rRNA gene. To secure a broad coverage of potential T2D associated bacteria, two technologies were used: The GA-map® 131-plex, utilizing 131 DNA probes complementary to pre-selected bacterial targets (covering the 16S regions V3-V9), and the LUMI-Seq™ platform, a full-length 16S sequencing technology (V1-V9). Variations in the gut microbiota between groups were explored using multivariate methods, differential bacterial abundance was estimated, and microbiota signatures discriminating the groups were assessed using classification models.

**Results:**

In total, 24 pre-T2D patients, 18 T2D patients, and 52 healthy subjects were recruited. From the LUMI-Seq™ analysis, 10 and 9 bacterial taxa were differentially abundant between pre-T2D and healthy, and T2D and healthy, respectively. From the GA-map® 131-plex analysis, 10 bacterial markers were differentially abundant when comparing pre-T2D and healthy. Several of the bacteria were short-chain fatty acid (SCFA) producers or typical opportunistic bacteria. Bacteria with similar function or associated properties also contributed to the separation of pre-T2D and T2D from healthy as found by classification models. However, limited overlap was found for specific bacterial genera and species.

**Conclusions:**

This pilot study revealed that differences in the abundance of SCFA producing bacteria, and an increase in typical opportunistic bacteria, may contribute to the variations in the microbiota separating the pre-T2D and T2D patients from healthy subjects. However, further efforts in investigating the relationship between gut microbiota, diabetes, and associated factors such as BMI, are needed for developing specific diabetes microbiota signatures.

## Background

Type 2 diabetes (T2D) is a significant global health challenge, constituting over 90% of diabetes cases worldwide, and with more than 700 million adults estimated to be impacted by 2045 [1]. Often there is an extended pre-diagnostic period, and a large proportion of people with T2D are thought to be undiagnosed. T2D pathophysiology involves gradually rising blood glucose levels (hyperglycemia) due to increasing insulin resistance and/or decreasing beta cell function, and is strongly associated with overweight and obesity and/or central adiposity [1, 2]. Prediabetic individuals have blood glucose levels higher than normal but below the threshold for T2D, are often overweight, and have an elevated risk of T2D and cardiovascular disease.

Gut microbiota plays a pivotal role in metabolism, immunomodulation and overall human health, and disruptions to the balance of this community are associated with many diseases, including inflammatory bowel disease (IBD) and irritable bowel syndrome (IBS), and metabolic diseases such as T2D [3]. Abundant evidence in both animal models and humans with T2D point to an altered or dysbiotic gut microbiota composition as compared to that found in healthy individuals [4–9]. Individuals with an unbalanced gut microbiota composition may exhibit a plethora of detrimental health outcomes: higher BMI, increased fat mass, reduced insulin sensitivity, dyslipidemia, and an increased inflammatory state [10]. It has been proposed that the intestinal bacteria and the metabolites they produce play a role in inducing a harmful chronic low-grade inflammatory state and subsequent development of insulin resistance [3, 9]. However, so far, no typical microbiota profile for T2D has been identified.

Over the last decade, the most common approach for microbiota profiling has been to target the nine hypervariable regions (V1–V9) of the bacterial 16S rRNA gene. This relatively short (∼1,500 bp) gene region provides phylogenetic signatures on different taxonomic levels. The hypervariable regions are surrounded by highly conserved sequences, which are used for primer design.

The aim of this pilot study was to explore the gut microbiota and bacteria associated with pre-T2D patients, and newly diagnosed, treatment naïve T2D patients, compared to healthy subjects. To secure a broad coverage of potential T2D associated bacteria, two technologies were used: The GA-map® technology platform 131-plex (GA-map® 131-plex), utilizing 131 DNA probes complementary to pre-selected bacterial targets (covering the 16S rRNA gene regions V3-V9), and the Long 16S using Unique Molecular Identifiers – Sequencing (LUMI-Seq™) platform, a full-length 16S sequencing technology (V1-V9).

## Methods

### Study population and sample collection

Adult patients diagnosed with either pre-T2D (*n* = 24) or T2D (*n* = 18) were recruited, and native fecal samples collected, by three diabetes clinics in Norway (Østerås, Sandnes and Tananger). In addition, healthy adult subjects (*n* = 52) were recruited, and native fecal samples collected by Oslo Metropolitan University (OsloMet) in Oslo, Norway. The study was approved by the regional Norwegian ethical committee (REC South-East, Norway), and informed consent was obtained from all participants. All samples and information were de-identified before analysis. The samples collected by the clinics were sent non-frozen to Genetic Analysis (GA), Oslo, Norway by mail and frozen upon delivery. The samples collected by OsloMet were frozen upon delivery to the university, before transferal to GA (frozen, on dry ice). All fecal samples were frozen (between -40 °C and -80 °C) within 5 days after collection before further processing.

A case record form was completed for all participants. The T2D patients were all newly diagnosed and treatment-naïve, except for one patient (> 5 months since the last dose of metformin). Criteria for the inclusion of T2D patients included analysis of blood glucose levels, specifically, hemoglobin A1c (HbA1c) ≥ 6.5%. Inclusion criteria for the pre-T2D patients included HbA1c of 6.0–6.4%. Criteria for the inclusion of healthy subjects included no history of diabetes or pre-diabetes, and HbA1c < 6.0%. Exclusion criteria for all groups included recent use of antibiotics (last 4 weeks), and a positive fecal calprotectin (F-cal) test (> 200 mg/kg). See Table 1 for characteristics of the study population. Samples from 86 subjects (16 T2D, 22 pre-T2D and 48 healthy) were analyzed with LUMI-Seq™ and included in the downstream data analysis (4 healthy, 2 pre-T2D and 2 T2D excluded after sequencing due to low taxa counts). Samples from 78 subjects (18 T2D, 22 pre-T2D and 38 healthy) were analyzed with the GA-map® 131-plex, after exclusions (11 healthy subjects excluded due to lack of sufficient number of wells in the plate setup, and additional 5 subjects (2 pre-T2D, 3 healthy) due to F-cal > 200 mg/kg).

**Table 1:** Study population – main characteristics

**Characteristics**	**Type 2 Diabetes**^**a**^^**,b**^		**Pre-Type 2 Diabetes**^**a,b**^		**Healthy**^**a,b**^	
	**LUMI-Seq (*n* = 16)**	**131-plex (*n* = 18)**	**LUMI-Seq (*n* = 22)**	**131-plex (*n* = 22)**	**LUMI-Seq (*n* = 48)**	**131-plex (*n* = 38)**
Male/female (*n*)	13/3	14/4	12/10	13/9	12/36	8/30
Age, median	64 (54–72)	64 (53–71)	67 (53–74)	66 (53–74)	31 (26–50)	40 (26–51)
BMI, median	28 (26–32)	28 (26–33)	30 (27–34)	30 (26–34)	23 (21–25)	23 (22–25)
HbA1c, %, median	6.8 (6.6–7.2)	6.8 (6.6–7.6)	6.1 (6.1–6.2)	6.1 (6.0–6.2)	5.2 (5.0–5.5)	5.2 (5.0–5.5)
F-cal, mg/kg, median	31 (12–68)	31 (13–61)	50 (32–119)	46 (32–89)	27 (15–55)	23 (14–45)

The characteristics of the participants in each of the study groups, analyzed and included in the LUMI-Seq™ and GA-map® 131-plex data analysis (the 25^th^ and 75^th^ percentiles are shown in parentheses)

^a^Samples from 16 T2D, 22 pre-T2D and 48 healthy were analyzed with LUMI-Seq™

^b^Samples from 18 T2D, 22 pre-T2D and 38 healthy were analyzed with the GA-map® 131-plex

### Sample processing and analysis

**Fig. 1:**
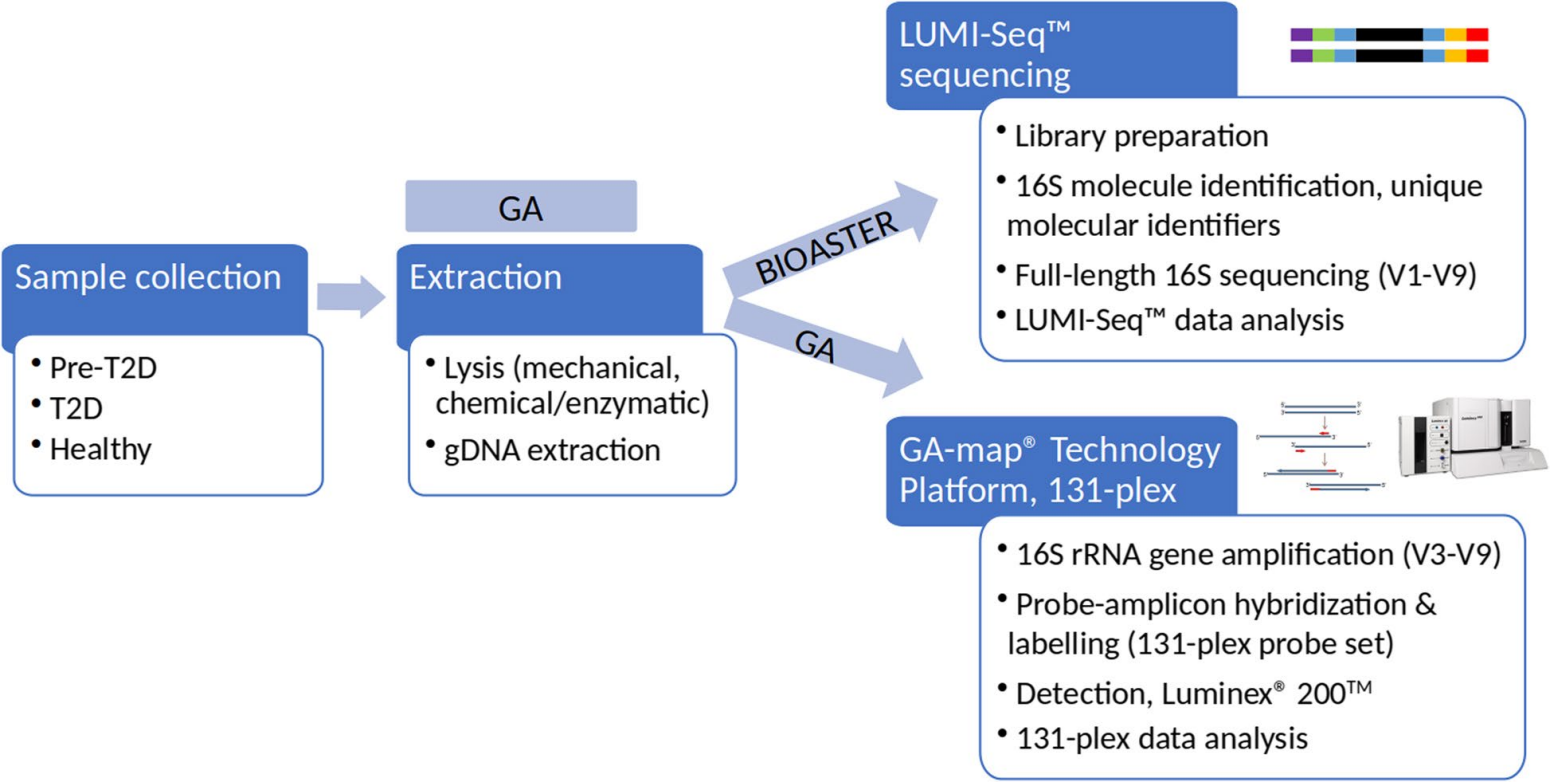
Study workflow. Starting with collection of fecal samples, and genomic DNA extraction at Genetic Analysis (GA), through analysis of the DNA samples on the two different platforms: LUMI-Seq™ (Long 16S using Unique Molecular Identifiers – Sequencing) at BIOASTER and the GA-map® Technology Platform 131-plex at GA

Total bacterial genomic DNA extraction was performed by GA, using a protocol previously described [11]. Briefly, the extraction was performed by fecal homogenization (using stirring rod) and mechanical cell lysis (FastPrep-96™, MP Biomedicals), followed by chemical/enzymatic heat lysis and automated DNA extraction using a MagMAX™ Express-96 or KingFisher™ Flex (Thermo Fisher Scientific) in combination with the mag™ maxi reagent kit (LGC Genomics GmbH). After extraction, DNA samples were aliquoted, and aliquots were shipped to BIOASTER in Lyon, France. The DNA samples were analyzed using the LUMI-Seq™ platform (BIOASTER, France) and by the GA-map® 131-plex (Genetic Analysis, Norway) (Fig. 1).

#### GA-map® 131-plex (GA-map® Technology Platform 131-plex)

The GA-map® 131-plex utilizes a pre-targeted approach, based on DNA probe hybridization to bacterial 16S rRNA gene targets, to identify and characterize bacterial profiles from fecal samples. This research-only panel of DNA probes (bacterial markers) was established to cover major bacterial observations made from the literature relating to the microbiota in healthy, IBS and IBD [11]. Disruptions to the regular community of bacteria have also been associated with other conditions, such as diabetes type 2 [3]. Each bacterial marker was designed to identify a specific bacterial species or group (e.g., phylum, class, genus), based on their 16S rRNA gene sequence [11]. As such, a large number of bacteria are detected at different taxonomic levels. The bacterial markers were intensively tested *in-silico* (target detection, non-target exclusion, cross‐labelling, self-labelling, and cross hybridization), and against bacterial DNA from a selection of culturable bacterial species in vitro.

The same laboratory procedures as described for the standardized and CE-marked GA-map® technology platform Dysbiosis Test were followed [11], with a few modifications (Fig. 1). Briefly (after DNA extraction), the 16S rRNA gene hypervariable regions V3-V9 are amplified by the polymerase chain reaction (PCR) using a universal primer pair [11]. The amplified DNA is hybridized to a 131-plex panel of DNA probes, complementary to regions within the amplicon specific for the targeted bacteria. Hybridized probes are labeled with biotin through single nucleotide extension before hybridization of the probe-set and solid-phase (carboxylated magnetic beads), as well as addition of a detection fluorophore. After washing of the samples, the fluorescent signal (probe signal intensity), corresponding to the abundance of target bacteria in the sample, is detected and quantified using a Luminex® 200™ instrument (Luminex Corp., Austin, TX, USA).

#### LUMI-Seq™ platform (Long 16S using Unique Molecular Identifiers—Sequencing)

Synthetic-long read sequencing is now emerging in the microbiome space as a methodology for generation of reliable quality long fragments from Illumina short reads [12–14]. In that context, BIOASTER recently developed LUMI-Seq™ to recover thousands of full-length 16S sequences from complex samples [15]. For this study, the standard LUMI-Seq™ workflow was followed (Fig. 1), in which each 16S molecule within each sample was first barcoded using unique molecular identifiers (UMI) for their tracking during the entire workflow. Then, the molecules were amplified to make multiple copies and to increase the signal. The PCR products were then fragmented while keeping the UMI information on all pieces. The fragments were sequenced using the MiSeq™ platform (Illumina, San Diego, California), in 2 × 200 bp. Preprocessing of the raw data was performed to remove low-quality ends of the reads with fastp [16]. Read pairs sharing the same UMI and the same sample barcode were grouped together in silico to make long accurate consensus sequences. Assembly was performed with SPAdes [17]. The reconstruction of full-length 16S sequences was performed using V-Revcomp [18] and V-Xtractor [19]. On average, 4,812 full-length 16S sequences were reconstructed per sample (range: 1,124–10,511). Based on the UMI redundancy, the LUMI-Seq™ error rate was assessed at 0.0047%.

### Statistical analysis

#### GA-map® 131-plex data analysis

To account for variable signal levels, the raw signal data (fluorescent intensities) was normalized using a hybridization control, as previously described [11], and background noise was subtracted. Variations in the bacterial profiles between and within the groups (pre-T2D, T2D and healthy) were explored using the non-parametric multivariate methods principal component analysis (PCA) and permutational multivariate analysis of variance (PerMANOVA), using Euclidean and Bray–Curtis methods, respectively. Possible confounding effects on the data due to the clinical variables (e.g., age, BMI, F-cal) were also explored, using the above-mentioned methods. The non-parametric Wilcoxon Rank Sum Test with Benjamini–Hochberg correction (Stats R package [20]) was used to determine significant difference (adjusted *p* < 0.1) in abundance of the bacterial markers.

Microbiota signatures for separation of pre-T2D or T2D and healthy subjects were calculated using the caret (classification and regression training) R-package [20]. To evaluate the robustness and performance of the classification models, a tenfold cross-validation was performed. 90% of the cohort was used for model training and 10% for model testing. A parameter, “importance”, ranging from 0 (no contribution) to 100 (maximum possible contribution for training of the model), was reported for each bacterial marker forming the basis for the microbiota signatures. The best performing model, multi-step adaptive elastic-net (MSA-ENet) [21], was chosen. A ROC curve was built, presenting the mean value of the area under the curve (AUC).

#### LUMI-Seq™ data analysis

After obtaining full-length 16S sequences, QIIME scripts (version 2019.7.0) were used for collapse of 100% identical sequences [22]. Each unique sequence was then assigned to a taxonomy by mapping to a custom 16S database made by BIOASTER, as well as the widely used SILVA reference database. However, due to the lower number of sequences assigned down to the species level, the BIOASTER database was chosen for the downstream analyses (76% with the 16S database vs. 60% with SILVA). Taxa with a total count (summed over all samples) lower than five counts were removed. After normalization of taxa counts, eight samples (four healthy, two pre-T2D and two T2D) showed lower counts and were thus discarded from the analysis to avoid interpretation bias.

Principal coordinates analysis (PCoA) and PerMANOVA, using the Bray–Curtis method, was used to explore variations in bacterial profiles between and within the groups (pre-T2D, T2D and healthy subjects). The above-mentioned methods were also used to explore any confounding effects on the data due to the clinical variables (e.g., age, sex, BMI). Differential abundance analyses were conducted with the DESeq2 package from Bioconductor [23, 24]. Based on a Wald test, taxa with an absolute log-fold change larger than a 0.5 threshold and an adjusted *p*-value lower than 0.05 were considered as differentially abundant.

Microbiota signatures for the separation of pre-T2D or T2D and healthy subjects were calculated using one of the most commonly used classifiers, Random Forest [25], in a fivefold cross-validation. The sample assignation was repeated until the three groups were equally distributed in the 5-fold range based on the Fisher’s exact test. As the majority of the taxa and genes were not associated with the groups, a univariate selection (Welch test, *p*-value < 1% or 10%) was performed to reduce the dimensionality. The cross-validation procedure was repeated 300 times.

## Results

### Multivariate analysis of the bacterial profiles

**Fig. 2:**
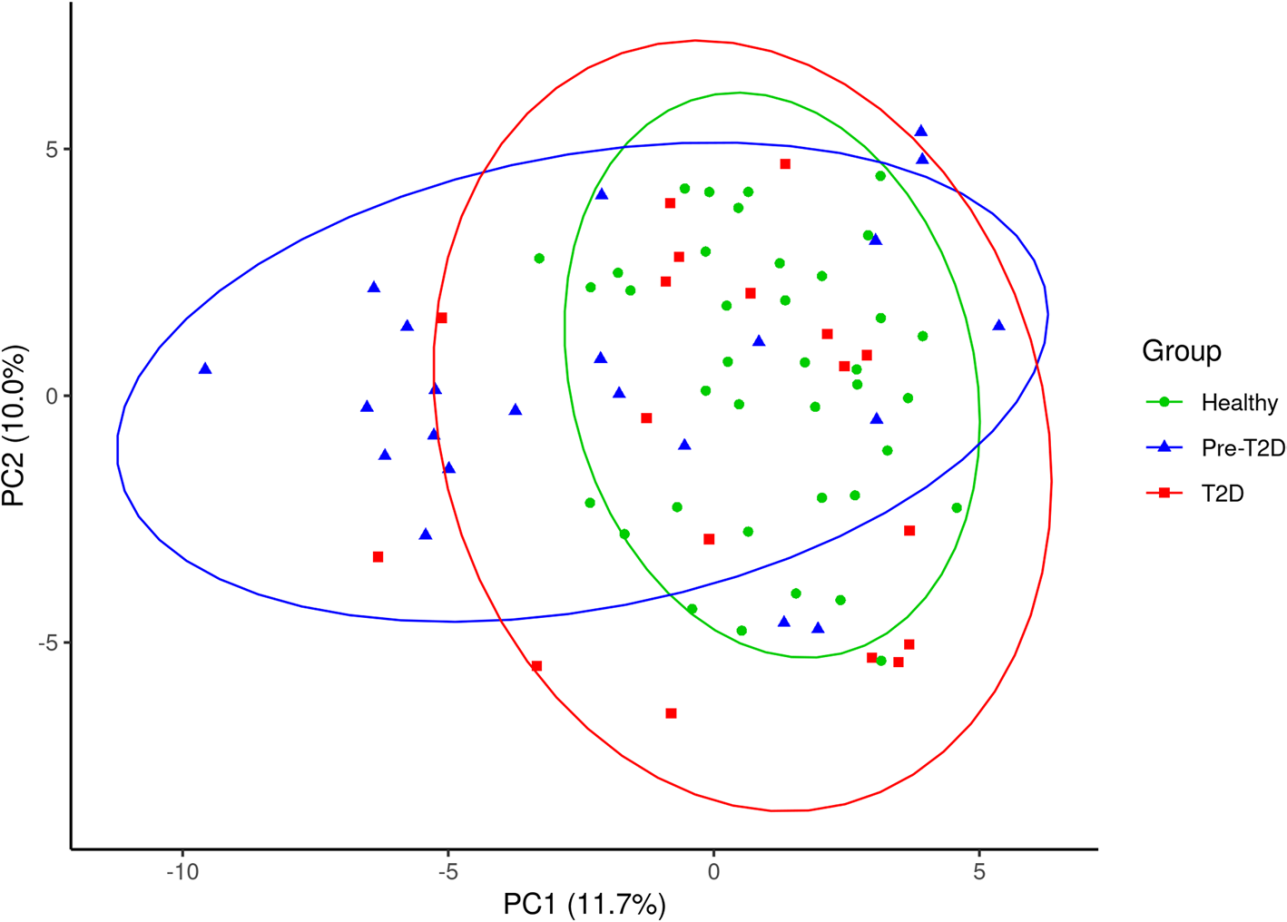
Principal Component Analysis (PCA) of GA-map® 131-plex data. The PCA score plot illustrates the similarities and variations of the groups healthy (*n* = 38), pre-T2D (*n* = 22) and T2D (*n* = 18), based on scaled and log-transformed normalized signal strength data. 90% confidence ellipses are shown for each of the groups

For the GA-map® 131-plex data, the PCA score plot (Fig. 2), indicates the largest differences in the bacterial profiles of the pre-T2D group as compared to the healthy group. Additionally, PerMANOVA analysis revealed significant impact of BMI (*p*: 0.001, *R*^2^: 0.93) on the data, while the parameters clinical groups (*p*: 0.001, *R*^2^: 0.063), age (*p*: 0.8, R^2^: 0.56), sex (*p*: 0.038, *R*^2^: 0.022) and F-cal (*p*: 0.106, *R*^2^: 0.67), had little to no significant impact. For the LUMI-Seq™ data, PCoA revealed little discrimination of samples according to the clinical groups (data not shown). Further, PerMANOVA analysis showed no significant impact on the data of the parameters clinical groups (*p*: 0.168, *R*^2^: 0.03), age (*p*: 0.297, *R*^2^: 0.01), sex (*p*: 0.23, *R*^2^: 0.01) and BMI (*p*:0.053, *R*^2^: 0.02), tested as one PerMANOVA, without interaction.

### Differential abundance analysis and microbiota signatures

**Fig. 3:**
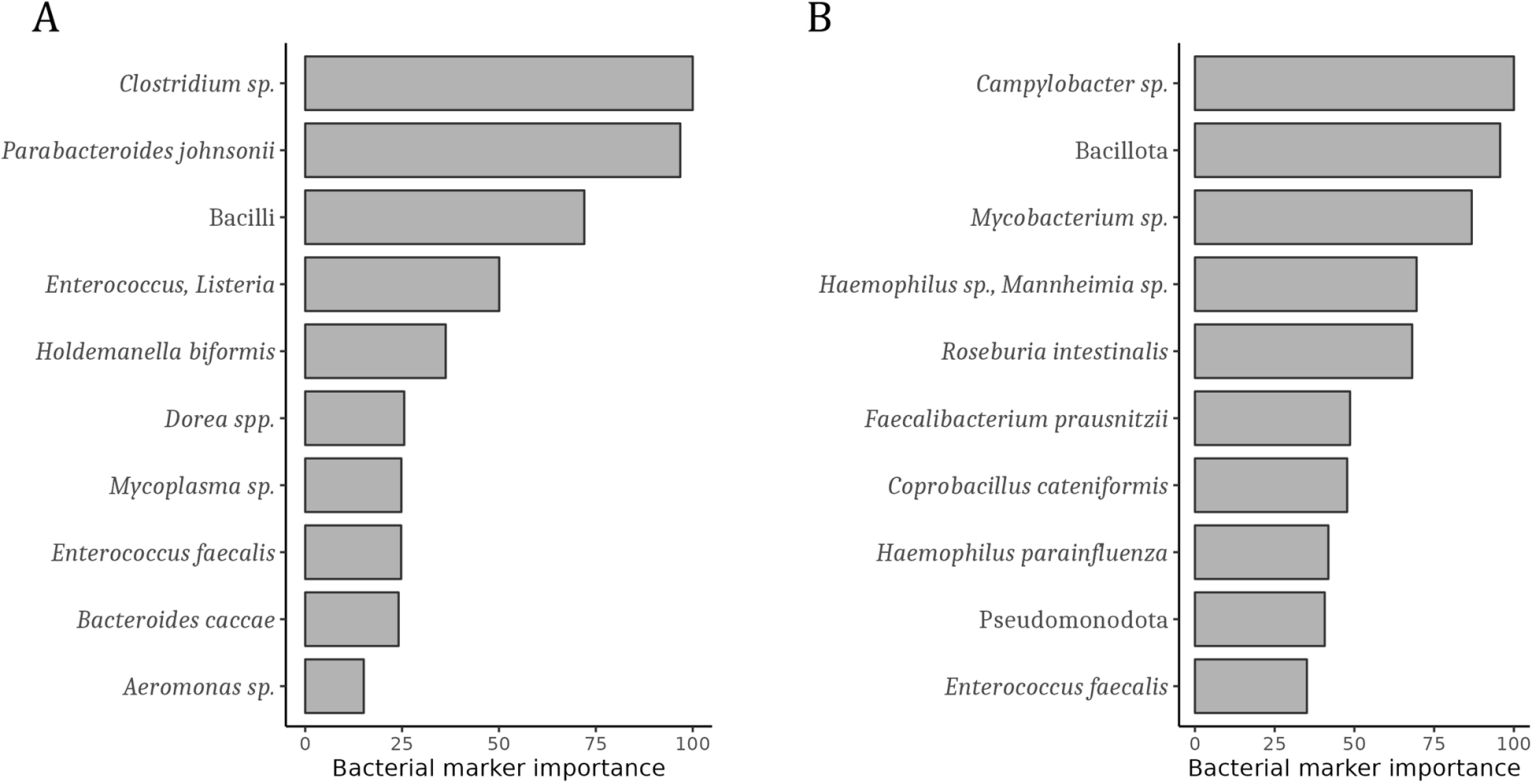
GA-map® 131-plex classification. The 10 most discriminant bacterial markers for separation of the groups **A**) pre-T2D (*n* = 22) and healthy (*n* = 38), or **B**) T2D (*n* = 18) and healthy (*n* = 38), based on microbiota signatures. Measured by ‘Bacterial marker importance’ as computed by MSA-ENet classifier

**Fig. 4:**
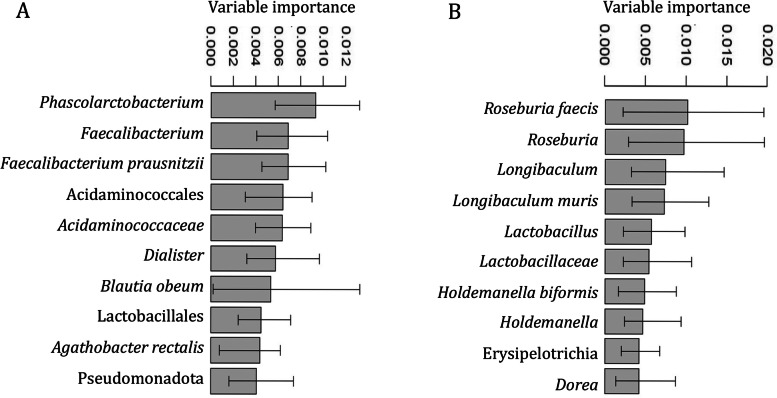
LUMI-Seq™ classification. The 10 most discriminant bacterial taxa for separation of the groups **A**) pre-T2D (*n* = 22) and healthy (*n* = 48), or **B**) T2D (*n* = 16) and healthy (*n* = 48), based on microbiota signatures. Measured by ‘Variable importance’ as computed by Random Forest classifier

As found by the combined results from the GA-map® 131-plex and LUMI-Seq™ data analysis, mainly bacteria from the phyla Bacillota (Firmicutes), were differentially abundant in pre-T2D and T2D compared to the healthy group (Tables 2 and 3), also confirmed by classification modelling (Figs. 3A&B and 4A&B). Note that from the GA-map® 131-plex analysis, no significant abundance differences were found when comparing the T2D group and the healthy group.

**Table 2:** Differentially abundant bacteria – pre-T2D vs. healthy, GA-map® 131-plex

**Groups**	**Phylum**	**Class**	**Bacterial marker**	**I/D**^**a**^	***p* adj**
Pre-T2D vs. healthy	Bacillota (Firmicutes)	Clostridia	*Dorea* spp.	I	9.30E-02
			*Roseburia* sp.	D	9.30E-02
			*Roseburia intestinalis*	D	9.30E-02
			*Faecalibacterium prausnitzii*	D	3.90E-02
		Negativicutes	*Dialister invisus*	D	9.30E-02
			*Veillonella* spp.	D	8.20E-02
	Pseudomonadota (Proteobacteria)	(Phylum marker)	Pseudomonadota	I	2.00E-02
		Gammaproteobacteria	Enterobacterales	I	3.20E-02
			*Shigella* spp. & *Escherichia* spp.	I	6.90E-02
		Betaproteobacteria	*Sutterella wadsworthensis*	D	9.30E-02

Bacterial markers with significantly different abundance (adjusted *p* < 0.1) when comparing the groups pre-T2D (*n* = 22) and healthy (*n* = 38), based on the GA-map® 131-plex data

^a^Increased (I) or decreased (D) abundance, in pre-T2D (vs. healthy)

**Table 3:** Differentially abundant bacteria — pre-T2D and T2D vs. healthy, LUMI-Seq™

**Groups**	**Phylum**	**Class**	**Bacterial taxa**	**Log2Fold Change**^**a**^	***p* adj**
Pre-T2D vs. healthy	Bacillota (Firmicutes)	Bacilli	Bacilli	1.84	2.42E-04
			Lactobacillales	1.90	2.42E-04
			*Streptococcaceae*	1.76	4.03E-04
			*Streptococcus*	1.92	2.42E-04
		Clostridia	*Eisenbergiella*	1.86	1.20E-03
			*Eisenbergiella massiliensis*	1.43	1.25E-02
			*Neglecta*	1.35	4.03E-04
			*Neglecta timonensis*	1.35	4.03E-04
			*Oscillibacter valericigenes*	-0.77	3.26E-02
	Bacteroidota (Bacteroidetes)	Bacteroidia	*Bacteroides coprocola*	-23.36	2.90E-17
T2D vs. healthy	Bacillota (Firmicutes)	Clostridia	*Dorea*	1.16	1.22E-02
			*Dorea formicigenerans*	0.89	3.14E-02
			*Dorea longicatena*	1.03	2.34E-02
			*Anaerotignum*	-0.99	3.14E-02
		Erysipelotrichia	Erysipelotrichia	1.05	1.91E-02
			Erysipelotrichales	1.05	1.91E-02
			*Erysipelotrichaceae*	1.05	1.91E-02
			*Turicibacter*	0.71	3.56E-02
			*Turicibacter sanguinis*	0.71	3.56E-02

Bacterial taxa with significantly different abundance (adjusted *p* < 0.05) when comparing the groups pre-T2D (*n* = 22) and healthy (*n* = 48), or T2D (*n* = 16) and healthy (*n* = 48), based on the LUMI-Seq™ data

^a^Increased or decreased abundance, in pre-T2D (vs. healthy), or in T2D (vs. healthy)

For instance, according to the GA-map® 131-plex data analysis (Table 2), the Clostridia *Faecalibacterium prausnitzii* and *Roseburia intestinalis*, major producers of the SCFA butyrate [26], and the Negativicutes *Veilonella* spp. and *Dialister (D. invisus)*, that can produce SCFAs such as propionate and acetate [27, 28], were more abundant (adjusted *p* < 0.1) in healthy than pre-T2D. From the classification modeling, the butyrate producing *R. intestinalis* and *F. prausnitzii* [26] were also among the top ten contributors for the separation of T2D and healthy (Fig. 3B), while the Bacteroidota *Parabacteroides johnsonii*, producer of the SCFAs succinate and acetate [29], contributed to the separation of pre-T2D and healthy (Fig. 3A). Based on the LUMI-Seq™ classification modeling, *F. prausnitzii* and another major butyrate producer, *Agathobacter rectalis* (*Eubacterium rectale*) [26], and the Negativicutes *Phascolarctobacterium* and *Dialister*, that can produce e.g., propionate and acetate [30, 28], were among the top ten contributors for separation of pre-T2D and healthy (Fig. 4A). The butyrate producers *Roseburia* and *Roseburia faecis* [26] also contributed to the separation of T2D and healthy (Fig. 4B).

Oppositely, e.g., the Clostridia *Dorea* spp., gas- and SCFA-producing bacteria [31], was more abundant in pre-T2D according to the GA-map® 131-plex analysis (Table 2), and, based on classification modeling, *Dorea* spp. contributed to the separation of pre-T2D and healthy (Fig. 3A). Similarly, as found from the LUMI-Seq™ analysis, the gas- and SCFA-producing *Dorea formicigenerans* and *D*. *longicatena* [31] were more abundant (adjusted *p* < 0.05) in T2D (Table 3), and *Dorea* was among the top ten contributors for the separation of T2D and healthy according to classification modeling (Fig. 4B).

Moreover, as found by the LUMI-Seq™ analysis (Table 3), *Erysipelotrichaceae*, possible SCFA producers also associated with metabolic disorders and inflammation [32, 33], and *Turicibacter sanguinis* (Erysipelotrichia class), a lactate producing bacteria [34], were more abundant in T2D. According to classification modeling, the possible SCFA producing Erysipelotrichia [32], including *Holdemanella biformis* (*Eubacterium biforme*)*,* contributed to the separation of T2D and healthy (Fig. 4B). *H. biformis*, that can produce SCFAs such as propionate and butyrate [35], also contributed to the separation of pre-T2D and healthy based on the GA-map® 131-plex classification modeling (Fig. 3A).

Further, based on the LUMI-Seq™ analysis (Table 3), Bacilli, including the lactic acid producing *Streptococcus* (Lactobacillales order), numerous being opportunistic [36], as well as *Eisenbergiella massiliensis* (Clostridia class), a potential SCFA producer that may be associated with obesity [37, 38], and *Neglecta timonensis* (Clostridia class), possibly associated with T2D [39], were more abundant in pre-T2D than in healthy. According to classification modeling, Lactobacillales, that may be both commensal and opportunistic [40], was among the top ten contributors for separation of pre-T2D and healthy (Fig. 4A), and the lactate producing *Lactobacillus* [41] for the separation of T2D and healthy (Fig. 4B). From the GA-map® 131-plex classification modeling, Bacilli (including *Enterococcus faecalis*), a diverse bacterial class containing both commensal and opportunistic bacteria [40], contributed to the separation of pre-T2D and healthy (Fig. 3A), and *E. faecalis* (Lactobacillales order)*,* as well as Bacillota, a highly diverse and abundant group of gut bacteria [40], to the separation of T2D and healthy (Fig. 3B).

Additionally, the opportunistic pro-inflammatory bacteria Pseudomonadota (Proteobacteria) and *Shigella* spp./*Escherichia* spp. (Gammaproteobacteria class) [42] were more abundant in pre-T2D according to the GA-map® 131-plex analysis (Table 2). Based on classification modeling, the Gammaproteobacteria *Aeromonas* sp., and the Bacillota *Clostridium* sp*.*, a potential pathogen [40], were among the top ten contributors for separation of pre-T2D and healthy (Fig. 3A), while Pseudomonadota, *Campylobacter* sp. (Epsilonproteobacteria), typical pathogenic bacteria [43], and the Gammaproteobacteria *Haemophilus* sp./*Mannheimia* sp., contributed to the separation of T2D and healthy (Fig. 3B). Pseudomonadota are also found by the LUMI-Seq™ classification to be among the top ten contributors for the separation of pre-T2D and healthy (Fig. 4A).

For the GA-map® 131-plex classification modeling, area under the curve (AUC) values of 0.88 or 0.77 were achieved for the separation of pre-T2D and healthy subjects, or T2D and healthy subjects, respectively. For the LUMI-Seq™ classification modeling, AUC values of 0.78 or 0.64 were achieved for the separation of pre-T2D and healthy subjects, or T2D and healthy subjects, respectively.

## Discussion

The primary aim of this pilot study was to explore the gut microbiota and associated bacteria in pre-T2D and treatment naïve T2D patients, compared to healthy subjects. LUMI-Seq™ and the GA-map® 131-plex represent two methods which can be used for bacterial profiling and to identify bacterial biomarkers. The results represent a preliminary discovery of possible diabetes specific bacterial patterns.

Differences in the abundance of SCFA producing bacteria in the phylum Bacillota (Firmicutes) between healthy subjects and pre-T2D and T2D patients were revealed. SCFA producing Bacillota were also among the top ten contributors for the separation of pre-T2D and T2D from healthy using classification models. For example, the SCFA producing bacteria *F*. *prausnitzii* and *Roseburia* were found to be less abundant, and the gas- and SCFA-producing *Dorea* more abundant, in pre-T2D and/or T2D, and were also among the ten most discriminative bacteria separating the groups from healthy. Additionally, SCFA producing bacteria such *A. rectalis (Eubacterium rectale)* and *H. biformis* (*Eubacterium biforme*), were indicated by classification models to contribute to this separation.

Further, also typical opportunistic bacteria contributed to the differentiation between the groups. For instance, bacteria from the Bacilli class, such as the opportunistic *Streptococcus*, and typical opportunistic, pro-inflammatory bacteria from the phylum Pseudomonadota (Proteobacteria) were increased in pre-T2D, and as found by classification models, contributed to the separation of pre-T2D and T2D from healthy.

*Roseburia, F. prausnitzii* and *A. rectalis* are major SCFA producing bacteria, particularly of butyrate, which have been negatively associated with T2D and/or pre-T2D [44–47]. Oppositely, *Dorea*, and bacteria from the Bacilli class and phylum Pseudomonadota have been found increased in T2D and/or pre-T2D [5, 6, 44, 45, 48]. Interestingly, *Dorea* may promote inflammation, and increased abundance of *Dorea* in T2D patients has also been negatively correlated with the abundance of butyrate-producing bacteria [49, 50].

Bacteria and their microbial products can impact the development of T2D by various and connected mechanisms, for instance, by affecting gut permeability, inflammatory regulation, and glucose metabolism (reviewed in [4, 8]). Butyrate producing bacteria and the metabolite butyrate are important for promoting anti-inflammatory properties, and maintaining regular gut functions, and may also improve insulin resistance and glucose tolerance [51, 52]. In contrast, factors such as bacterial-derived lipopolysaccharide (LPSs), e.g., coming from Pseudomonadota, as well as the increased abundance of opportunistic bacteria in itself, can promote inflammation, and may contribute to the induction of a low-grade inflammatory state and insulin resistance [53, 54].

A strength of this study is that DNA was extracted by the standardized GA-map® method, and DNA samples split, before analysis by the two analysis platforms, as the choice of extraction method may influence the end results [55, 56]. Thus, the use of one extraction method enables easier comparisons of the downstream results. Also, the combination of mechanical and chemical lysis (as utilized by the GA-map® method), has been shown to enhance the extraction of both Gram-negative and -positive bacteria, and to increase bacterial DNA yields [55, 56].

Another strength is the inclusion of treatment naïve T2D patients, as it has been shown that the use of the common diabetes drug Metformin may affect the gut microbiota [57, 58]. Including prediabetic patients and treatment naïve T2D patients may make it more straightforward to understand the connections between the disease development and gut microbiota—by avoiding the effect of treatment or prolonged disease. Further, participants included in this study had not used antibiotics recently, also known to influence the gut bacterial composition [59, 60].

Even though the same criteria for age and BMI were used for the inclusion of patients and healthy subjects, the healthy subjects were younger and had lower BMI (and included more females). Also, the pre-T2D group had a slightly higher median F-cal, and the LUMI-Seq™ analysis included 5 subjects that should have been excluded due to F-cal > 200. While PerMANOVA of the GA-map® 131-plex data, showed that BMI had a significant effect on the data, F-cal levels did not. This is perhaps not surprising, as diabetes is strongly associated with higher BMI/overweight [1, 2].

The GA-map® 131-plex detects 131 DNA probes representing pre-selected 16S rRNA bacterial targets, while the LUMI-Seq™ platform entails full length 16S rRNA sequencing. Differences in the targeted 16S regions for the GA-map® 131-plex and LUMI-Seq™ (V3-V9 vs. V1-V9, respectively), as well as the selected targets and the pre-determined taxonomic levels of the GA-map® method, may lead to differences in the phylogenetic resolution. This may be one reason for the two method’s limited overlap in genera and species of potential T2D associated bacteria. For instance, *Turicibacter sanguinis*, found elevated in T2D by LUMI-Seq™, cannot be detected directly by the GA-map® 131 plex – however, may be covered by the broad Bacillota marker. Another limitation may be the use of different statistical methods, chosen to fit the dataset in question. For instance, for the GA-map® 131-plex data, bacterial abundances were considered as significantly different if *p*.adj. < 0.1, as a limit of 0.05 gave limited results.

It is critical for researchers to take into consideration the strengths and limitations of different platforms and choose a system appropriate for their experimental design. The GA-map® platform offers the advantage of a standardized method, utilizing a pre-selected target approach, allowing for a reduced assay turn-around time and less resource-demanding data analysis. At the level of genera, the GA-map® technology exhibit strong correlation to MiSeq amplicon sequencing [11]. Even though the LUMI-Seq™ follows similar protocols as the standard Illumina sequencing, it is difficult to assess differences due to technical variations since no comparative study has been performed. However, the low error rate and the high number of sequences assigned to the species level in this study illustrates that the LUMI-Seq™ technology constitutes a robust approach for microbiota profiling studies.

This pilot study focused on a limited number of Scandinavian (Norwegian) participants only, and so the results and interpretation should be taken with caution. The recruitment and inclusion of treatment naïve T2D patients is especially challenging due to, following standard guidelines, the limited time between diagnosis and start of treatment. The lower number of pre-T2D and T2D patients may have affected the outcome and can be one explanation for the pre-T2D group seemingly having the most distinct microbiota composition. Differences in diet may be another factor affecting the results, as no detailed description of the diet was recorded. To strengthen the foundation for developing bacterial signatures for Type 2 diabetes, future studies should be larger, international, multi-site studies, to account for variation in inter-individual microbiota. Possible confounding factors that ought to be controlled closely include medication-use, diet, and lifestyle [59–63].

Multiple studies have provided compelling evidence of an altered state in gut microbiota composition in pre-T2D and T2D individuals as compared to healthy subjects, with a strong correlation to insulin resistance and β-cell dysfunction, detected even prior to glucose abnormalities in these individuals [46, 64–66]. The implication of an altered gut microbiota composition in diabetic and prediabetic patients was also supported by this study.

## Conclusions

This pilot study revealed that differences in the abundance of short chain fatty acid (SCFA) producing bacteria, and an increase in typical inflammation-associated or potentially pro-inflammatory or opportunistic bacteria, may contribute to the variations in the microbiota separating the pre-T2D and T2D patients from the healthy subjects. However, further efforts in investigating the relationship between gut microbiota, diabetes, and associated factors such as BMI, are needed for developing specific diabetes microbiota signatures.

## Data Availability

The sequence datasets (16S rRNA sequences) generated and/or analysed during the current study are available in DDBJ/EMBL/GenBank under the accession KHUV00000000. The version described in this paper is the first version, KHUV01000000. The other datasets are available from the corresponding author on reasonable request.

## Abbreviations

T2D: Type 2 Diabetes
BMI: Body Mass Index
SCFA: Short Chain Fatty Acids
IBS: Irritable Bowel Syndrome
IBD: Inflammatory Bowel Disease
Pre-T2D: Pre-Type 2 Diabetes
HbA1c: Hemoglobin A1c
F-cal: Fecal Calprotectin
PCR: Polymerase Chain Reaction
UMI: Unique Molecular Identifier
PCA: Principal Component Analysis
PCoA: Principal Coordinates Analysis
PerMANOVA: Permutational Multivariate Analysis of Variance
MSA-ENet: Multi-Step Adaptive Elastic-Net
LPS: Lipopolysaccharides
OsloMet: Oslo Metropolitan University
GA: Genetic Analysis
LUMI-Seq™: Long 16S using Unique Molecular Identifiers Sequencing
GA-map® 131-plex: GA-map® Technology platform 131-plex

## References

[1] International Diabetes Federation. IDF Diabetes Atlas, 10th edition 2021. Available from: https://diabetesatlas.org/.

[2] American Diabetes Association (2010). Diagnosis and classification of diabetes mellitus. Diabetes Care.

[3] Scheithauer TPM, Rampanelli E, Nieuwdorp M, Vallance BA, Verchere CB, van Raalte DH (2020). Gut microbiota as a trigger for metabolic inflammation in obesity and type 2 diabetes. Front Immunol.

[4] Cunningham AL, Stephens JW, Harris DA (2021). Gut microbiota influence in type 2 diabetes mellitus (T2DM). Gut Pathog.

[5] Larsen N, Vogensen FK, van den Berg FW, Nielsen DS, Andreasen AS, Pedersen BK (2010). Gut microbiota in human adults with type 2 diabetes differs from non-diabetic adults. PLoS One.

[6] Sedighi M, Razavi S, Navab-Moghadam F, Khamseh ME, Alaei-Shahmiri F, Mehrtash A (2017). Comparison of gut microbiota in adult patients with type 2 diabetes and healthy individuals. Microb Pathog.

[7] Zhao L, Lou H, Peng Y, Chen S, Zhang Y, Li X (2019). Comprehensive relationships between gut microbiome and faecal metabolome in individuals with type 2 diabetes and its complications. Endocrine.

[8] Gurung M, Li Z, You H, Rodrigues R, Jump DB, Morgun A (2020). Role of gut microbiota in type 2 diabetes pathophysiology. EBioMedicine.

[9] Cani PD, Bibiloni R, Knauf C, Waget A, Neyrinck AM, Delzenne NM (2008). Changes in gut microbiota control metabolic endotoxemia-induced inflammation in high-fat diet-induced obesity and diabetes in mice. Diabetes.

[10] Le Chatelier E, Nielsen T, Qin J, Prifti E, Hildebrand F, Falony G (2013). Richness of human gut microbiome correlates with metabolic markers. Nature.

[11] Casen C, Vebo HC, Sekelja M, Hegge FT, Karlsson MK, Ciemniejewska E (2015). Deviations in human gut microbiota: a novel diagnostic test for determining dysbiosis in patients with IBS or IBD. Aliment Pharmacol Ther.

[12] Burke CM, Darling AE (2016). A method for high precision sequencing of near full-length 16S rRNA genes on an Illumina MiSeq. PeerJ.

[13] Karst S, Dueholm M, McIlroy S, Kirkegaard RH, Nielsen PH, Albertsen M (2018). Retrieval of a million high-quality, full-length microbial 16S and 18S rRNA gene sequences without primer bias. Nat Biotechnol.

[14] Deutscher AT, Burke CM, Darling AE, Riegler M, Reynolds OL, Chapman TA (2018). Near full-length 16S rRNA gene next-generation sequencing revealed Asaia as a common midgut bacterium of wild and domesticated Queensland fruit fly larvae. Microbiome.

[15] IRB BIOASTER. LUMI-Seq®: TECHNOLOGIES DESIGNED BY BIOASTER, H264 2020 STUK. YouTube. 2021. Available from: https://www.youtube.com/watch?v=ZU6ri4y2TM4.

[16] Chen S, Zhou Y, Chen Y, Gu J (2018). fastp: an ultra-fast all-in-one FASTQ preprocessor. Bioinformatics.

[17] Bankevich A, Nurk S, Antipov D, Gurevich AA, Dvorkin M, Kulikov AS (2012). SPAdes: a new genome assembly algorithm and its applications to single-cell sequencing. J Comput Biol.

[18] Hartmann M, Howes CG, Veldre V, Schneider S, Vaishampayan PA, Yannarell AC (2011). V-REVCOMP: automated high-throughput detection of reverse complementary 16S rRNA gene sequences in large environmental and taxonomic datasets. FEMS Microbiol Lett.

[19] Hartmann M, Howes CG, Abarenkov K, Mohn WW, Nilsson RH (2010). V-Xtractor: an open-source, high-throughput software tool to identify and extract hypervariable regions of small subunit (16S/18S) ribosomal RNA gene sequences. J Microbiol Methods.

[20] The R Foundation. The R Project for Statistical Computing. Available from: https://www.r-project.org/.

[21] Xiao N, Xu QS (2015). Multi-step adaptive elastic-net: reducing false positives in high-dimensional variable selection. J Stat Comput Simul.

[22] Bolyen E, Rideout JR, Dillon MR, Bokulich NA, Abnet CC, Al-Ghalith GA (2019). Reproducible, interactive, scalable and extensible microbiome data science using QIIME 2. Nat Biotechnol.

[23] Love MI, Huber W, Anders S (2014). Moderated estimation of fold change and dispersion for RNA-seq data with DESeq2. Genome Biol.

[24] Bioconductor. DESeq2. Available from: https://bioconductor.org/packages/release/bioc/html/DESeq2.html.

[25] Breiman L (2001). Random forests. Mach Learn.

[26] Louis P, Flint HJ (2009). Diversity, metabolism and microbial ecology of butyrate-producing bacteria from the human large intestine. FEMS Microbiol Lett.

[27] Duncan SH, Louis P, Flint HJ (2004). Lactate-utilizing bacteria, isolated from human feces, that produce butyrate as a major fermentation product. Appl Environ Microbiol.

[28] Wade WG. Dialister. Bergey's Manual of Systematics of Archaea and Bacteria. 2015;1–5. 10.1002/9781118960608.gbm00696.

[29] Sakamoto M, Kitahara M, Benno Y (2007). Parabacteroides johnsonii sp. nov., isolated from human faeces. Int J Syst Evol Microbiol.

[30] Stackebrandt E, Osawa R. Phascolarctobacterium. Bergey's Manual of Systematics of Archaea and Bacteria. 2015;1–4. 10.1002/9781118960608.gbm00700

[31] Taras D, Simmering R, Collins MD, Lawson PA, Blaut M (2002). Reclassification of Eubacterium formicigenerans Holdeman and Moore 1974 as Dorea formicigenerans gen. nov., comb. nov., and description of Dorea longicatena sp. nov., isolated from human faeces. Int J Syst Evol Microbiol.

[32] Wu J, Liu M, Zhou M, Wu L, Yang H, Huang L, Chen C (2021). Isolation and genomic characterization of five novel strains of Erysipelotrichaceae from commercial pigs. BMC microbiol.

[33] Kaakoush NO (2015). Insights into the role of Erysipelotrichaceae in the human host. Front Cell Infect Microbiol.

[34] Bosshard PP, Zbinden R, Altwegg M (2002). Turicibacter sanguinis gen. nov., sp. nov., a novel anaerobic, Gram-positive bacterium. Int J Syst Evol Microbiol.

[35] De Maesschalck C, Van Immerseel F, Eeckhaut V, De Baere S, Cnockaert M, Croubels S (2014). Faecalicoccus acidiformans gen. nov., sp. nov., isolated from the chicken caecum, and reclassification of Streptococcus pleomorphus (Barnes et al. 1977), Eubacterium biforme (Eggerth 1935) and Eubacterium cylindroides (Cato et al. 1974) as Faecalicoccus pleomorphus comb. nov., Holdemanella biformis gen. nov., comb. nov. and Faecalitalea cylindroides gen. nov., comb. nov., respectively, within the family Erysipelotrichaceae. Int J Syst Evol Microbiol.

[36] Willenborg J, Goethe R (2016). Metabolic traits of pathogenic streptococci. FEBS Lett.

[37] Amir I, Bouvet P, Legeay C, Gophna U, Weinberger A (2014). Eisenbergiella tayi gen. nov., sp. nov., isolated from human blood. Int J Syst Evol Microbiol.

[38] Togo AH, Khelaifia S, Bittar F, Maraninchi M, Raoult D, Million M (2016). 'Eisenbergiella massiliensis', a new species isolated from human stool collected after bariatric surgery. New Microbes New Infect.

[39] Bessis S, Ndongo S, Lagier JC, Raoult D, Fournier PE (2016). Neglecta timonensis' gen. nov., sp. nov., a new human-associated species. New Microbes New Infect.

[40] Rajilić-Stojanović M, de Vos WM (2014). The first 1000 cultured species of the human gastrointestinal microbiota. FEMS Microbiol Rev.

[41] Pot B, Felis GE, Bruyne KD, Tsakalidou E, Papadimitriou K, Leisner J, Vandamme P. The genus Lactobacillus. Lactic acid bacteria: biodiversity and taxonomy. 2014; 249–353. 10.1002/9781118655252.ch19.

[42] Rizzatti G, Lopetuso LR, Gibiino G, Binda C, Gasbarrini A (2017). Proteobacteria: a common factor in human diseases. Biomed Res Int.

[43] Moore JE, Corcoran D, Dooley JS, Fanning S, Lucey B, Matsuda M (2005). Campylobacter. Vet Res.

[44] Qin J, Li Y, Cai Z, Li S, Zhu J, Zhang F (2012). A metagenome-wide association study of gut microbiota in type 2 diabetes. Nature.

[45] Zhang X, Shen D, Fang Z, Jie Z, Qiu X, Zhang C (2013). Human gut microbiota changes reveal the progression of glucose intolerance. PLoS One.

[46] Wu H, Tremaroli V, Schmidt C, Lundqvist A, Olsson LM, Krämer M (2020). The gut microbiota in prediabetes and diabetes: a population-based cross-sectional study. Cell Metab.

[47] Tamanai-Shacoori Z, Smida I, Bousarghin L, Loreal O, Meuric V, Fong SB (2017). Roseburia spp.: a marker of health?. Future Microbiol.

[48] Pinna NK, Anjana RM, Saxena S, Dutta A, Gnanaprakash V, Rameshkumar G (2021). Trans-ethnic gut microbial signatures of prediabetic subjects from India and Denmark. Genome Med.

[49] Li Q, Chang Y, Zhang K, Chen H, Tao S, Zhang Z (2020). Implication of the gut microbiome composition of type 2 diabetic patients from northern China. Sci Rep.

[50] Schirmer M, Smeekens SP, Vlamakis H, Jaeger M, Oosting M, Franzosa EA (2016). Linking the human gut microbiome to inflammatory cytokine production capacity. Cell.

[51] Vrieze A, Out C, Fuentes S, Jonker L, Reuling I, Kootte RS (2014). Impact of oral vancomycin on gut microbiota, bile acid metabolism, and insulin sensitivity. J Hepatol.

[52] Vrieze A, Van Nood E, Holleman F, Salojärvi J, Kootte RS, Bartelsman JF (2012). Transfer of intestinal microbiota from lean donors increases insulin sensitivity in individuals with metabolic syndrome. Gastroenterology.

[53] Creely SJ, McTernan PG, Kusminski CM, Fisher FM, Da Silva NF, Khanolkar M (2007). Lipopolysaccharide activates an innate immune system response in human adipose tissue in obesity and type 2 diabetes. Am J Physiol Endocrinol Metab.

[54] Mehta NN, McGillicuddy FC, Anderson PD, Hinkle CC, Shah R, Pruscino L (2010). Experimental endotoxemia induces adipose inflammation and insulin resistance in humans. Diabetes.

[55] de Boer R, Peters R, Gierveld S, Schuurman T, Kooistra-Smid M, Savelkoul P (2010). Improved detection of microbial DNA after bead-beating before DNA isolation. J Microbiol Methods.

[56] Ma ZY, Zhang XM, Wang R, Wang M, Liu T, Tan ZL (2020). Effects of chemical and mechanical lysis on microbial DNA yield, integrity, and downstream amplicon sequencing of rumen bacteria and protozoa. Front. Microbiol.

[57] Forslund K, Hildebrand F, Nielsen T, Falony G, Le Chatelier E, Sunagawa S (2015). Disentangling type 2 diabetes and metformin treatment signatures in the human gut microbiota. Nature.

[58] Wu H, Esteve E, Tremaroli V, Khan MT, Caesar R, Mannerås-Holm L (2017). Metformin alters the gut microbiome of individuals with treatment-naïve type 2 diabetes, contributing to the therapeutic effects of the drug. Nat Med.

[59] Morgun A, Dzutsev A, Dong X, Greer RL, Sexton DJ, Ravel J (2015). Uncovering effects of antibiotics on the host and microbiota using transkingdom gene networks. Gut.

[60] Imhann F, Vich Vila A, Bonder MJ, Lopez Manosalva AG, Koonen DPY, Fu J (2017). The influence of proton pump inhibitors and other commonly used medication on the gut microbiota. Gut Microbes.

[61] Wu GD, Chen J, Hoffmann C, Bittinger K, Chen YY, Keilbaugh SA (2011). Linking long-term dietary patterns with gut microbial enterotypes. Science.

[62] Makki K, Deehan EC, Walter J, Bäckhed F (2018). The impact of dietary fiber on gut microbiota in host health and disease. Cell Host Microbe.

[63] David LA, Materna AC, Friedman J, Campos-Batista MI, Blackburn MC, Perotta A (2014). Host lifestyle affects human microbiota on daily timescales. Genome Biol.

[64] Allin KH, Tremaroli V, Caesar R, Jensen BAH, Damgaard MTF, Bahl MI (2018). Aberrant intestinal microbiota in individuals with prediabetes. Diabetologia.

[65] Zhou W, Sailani MR, Contrepois K, Zhou Y, Ahadi S, Leopold SR (2019). Longitudinal multi-omics of host-microbe dynamics in prediabetes. Nature.

[66] Tabak AG, Herder C, Rathmann W, Brunner EJ, Kivimaki M (2012). Prediabetes: a high-risk state for diabetes development. Lancet.

